# Neurobiology of Loneliness, Isolation, and Loss: Integrating Human and Animal Perspectives

**DOI:** 10.3389/fnbeh.2022.846315

**Published:** 2022-04-08

**Authors:** Erika M. Vitale, Adam S. Smith

**Affiliations:** Department of Pharmacology and Toxicology, School of Pharmacy, University of Kansas, Lawrence, KS, United States

**Keywords:** loneliness, isolation, loss, oxytocin, dopamine, CRH, opioids

## Abstract

In social species such as humans, non-human primates, and even many rodent species, social interaction and the maintenance of social bonds are necessary for mental and physical health and wellbeing. In humans, perceived isolation, or loneliness, is not only characterized by physical isolation from peers or loved ones, but also involves negative perceptions about social interactions and connectedness that reinforce the feelings of isolation and anxiety. As a complex behavioral state, it is no surprise that loneliness and isolation are associated with dysfunction within the ventral striatum and the limbic system – brain regions that regulate motivation and stress responsiveness, respectively. Accompanying these neural changes are physiological symptoms such as increased plasma and urinary cortisol levels and an increase in stress responsivity. Although studies using animal models are not perfectly analogous to the uniquely human state of loneliness, studies on the effects of social isolation in animals have observed similar physiological symptoms such as increased corticosterone, the rodent analog to human cortisol, and also display altered motivation, increased stress responsiveness, and dysregulation of the mesocortical dopamine and limbic systems. This review will discuss behavioral and neuropsychological components of loneliness in humans, social isolation in rodent models, and the neurochemical regulators of these behavioral phenotypes with a neuroanatomical focus on the corticostriatal and limbic systems. We will also discuss social loss as a unique form of social isolation, and the consequences of bond disruption on stress-related behavior and neurophysiology.

## Introduction

Social relationships are critical for health and wellbeing of the individual and for survival of social species. Thus, social behaviors are valued and motivating, while social isolation and ostracization are aversive and stressful. While different species exhibit a variety of behaviors that facilitate social bonding and maintain social relationships, many of the neural mechanisms that govern the display of these behaviors are conserved across species. For example, neuropeptides such as oxytocin (OT) and vasopressin, and neurotransmitters such as dopamine (DA) and endorphins, are critically involved in promoting affiliative and social-seeking behaviors. In addition, stress-related neuropeptides such as glucocorticoids and corticotrophin releasing hormone (CRH) are increased during social isolation and social ostracization, leading to anxiety- and depressive-like behaviors and altering sociality in various ways. This review will synthesize what we currently know about the neurobiological foundations of loneliness in humans and the use of animal models of social isolation to provide a more mechanistic approach to study how the brain adapts to being alone. Additionally, we will discuss social loss as a unique form of loneliness and isolation in both humans and animal models and how the neurochemical underpinnings of loss overlap with and diverge from those that influence loneliness in the absence of loss.

## Social Connectedness Is Rewarding and Stress Buffering in Social Species

From an ecological standpoint, species that have evolved to rely on sociality and community for survival should certainly have neurobiological mechanisms in place that drive individual members of the species to seek out and form relationships with others. In such species, social groups provide greater protection from predators (i.e., shared vigilance, mob defense), enhances success in locating and maintaining access to resources including food and mates, and increases survival of offspring by increasing the number of caregivers and protectors (Lee, 1994; Burkart and van Schaik, 2016; van Schaik, 2016). Socialization in these species begins early in development, and the social environment (and thus, the definition of what it means to be appropriately social at that time point) change throughout the lifespan (Box 1). Social interaction is driven neurobiologically by activation of reward circuitry involving the neurotransmitter DA being synthesized from the midbrain ventral tegmental area (VTA) and released into the ventral striatum (i.e., nucleus accumbens, NAc) and higher-order cortical regions (i.e., prefrontal cortex, PFC; insular cortex, IC; anterior cingulate cortex, ACC). Through this mechanism and via coordination with the neuropeptides OT and vasopressin that are released during social contact and intimacy, forming and maintaining social relationships is gratifying and valuable. Many social mammals show affiliation and affection through the physical touch of social grooming (Carter et al., 1999). In non-human mammals, social grooming causes a release of endogenous opioids (i.e., endorphins; Keverne et al., 1989), neurochemicals that promote reward and reduce pain sensitivity. This form of tactile stimulation is encoded by mechanoreceptive C-tactile (CT) fibers at the base of most hair follicles in many species (rodents, Leem et al., 1993; primates, Kumazawa and Perl, 1977; humans, Vallbo et al., 1999; Löken et al., 2009; Olausson et al., 2010). These axons are unmyelinated, respond specifically to slow and gentle stroking (∼2.5 cm per second), and directly trigger endorphin release (Nummenmaa et al., 2016). As humans no longer have fur to socially groom, these receptors respond instead to stroking, caressing, touching, and hugging which we use as a means of expressing and strengthening intimate relationships (Dunbar, 2010; Löken and Olausson, 2010; Suvilehto et al., 2015). Even non-tactile behaviors such as laughing (Caruana, 2017; Manninen et al., 2017), singing (Pearce et al., 2015), dancing (Tarr et al., 2015, 2016), and emotional story telling (Dunbar et al., 2016) in social contexts can facilitate the release of endogenous opioids. Thus, showing and receiving affection and intimacy activates neurobiological processes that reward and promote continued display of such behaviors.

**Box 1. Social development and loneliness across the lifespan.**

Social mammals experience a wide variety of social environments throughout their lifespan and navigate these social spaces with the skills they currently have at that developmental period. During infancy, the brain undergoes extensive plasticity including the making and breaking of connections via the growth of dendritic arborizations, synaptogenesis, and myelination to strengthen existing connections (Gao et al., 2009; Tau and Peterson, 2010). During this time, the social environment, which mainly consists of caregivers and siblings, can have drastic impacts on the development of early social behavior. For instance, rodent studies have shown that the amount and quality of maternal care influences adult maternal behavior, sexual behavior, and peer interactions (Bosch and Neumann, 2012). Additionally, when parental behavior is stable and predictable, infants (humans, non-human primates, rats) develop optimally (Johnson et al., 1996; Davis et al., 2017). Furthermore, developmental settings that are associated with unstable and unpredictable caregiving, such as postpartum psychopathology, loss of a caregiver, and orphanhood, interfere with social development. In support, infants of mothers with postpartum depression show disrupted attachment to their mother (McMahon et al., 2006; Lanzi et al., 2009) and lower social engagement (Feldman et al., 2009). Loss of one or more parental caregivers disrupts later social behavior and bonding in prairie voles (Rogers and Bales, 2020; Valera-Marín et al., 2021) and in non-human primates (Harlow and Suomi, 1971; Winslow et al., 2003), and it is estimated that between one half and two thirds of children entering the foster care system demonstrate emotional or behavioral problems (Chernoff et al., 1994). Parental insensitivity also predicts higher sympathetic nervous system activity in adult children during conflict discussions with their romantic partner (Raby et al., 2015), indicating that childhood social environment can have long-lasting impacts on future social behavior. As an extension, the parent–infant dyad helps infants learn the behavioral repertoire of sociality, including bonding, cooperation, and competition to promote a long-term framework for social affiliation. The social environment expands drastically once infants reach childhood and begin interacting with others of their age. Lack of high-quality peer relationships are also one of the first sources of loneliness, especially as children transition from forming and maintaining relationships based on proximity and shared interest to desiring relationships that have more positive qualities. Forming high-quality friendships with peers can positively impact social confidence and success navigating the social world (see Collins and Laursen, 1999; Hartup and Stevens, 1999); however, friendships with negative qualities, such as dominance and rivalry, can negatively impact children’s social skills, increase disruptive and disagreeable behaviors, and increase feelings of loneliness (Keefe and Berndt, 1996; Ladd et al., 1996). Exclusion from peer groups in general is also linked to loneliness in kindergarteners (Kochenderfer-Ladd and Wardrop, 2001). Adolescence is characterized by a further shift in prioritizing quality within social relationships, a desire to be liked by the entire peer group, and a new focus on intimacy within social relationships. Indeed, lack of friendship, low friendship quality, and peer rejection are all predictors of loneliness in adolescence (Vanhalst et al., 2015). Interestingly, stability of loneliness across childhood and adolescence is variable, with some individuals remaining high from age 7 to 17, some starting low and increasing over time, some starting high and decreasing over time, and some with low, stable levels of loneliness (Qualter et al., 2013). Additionally, negative reactivity, low levels of social engagement, and low social preference increased the risk of following the high loneliness trajectory. Romantic relationships also emerge as a new source of loneliness during this time (Collins et al., 2009) and quality of romantic relationships has a lasting impact on loneliness. For instance, relational disappointment is associated with higher levels of loneliness in college students (Flora and Segrin, 2000), and marriage quality explains individual differences in loneliness in adulthood (Hawkley et al., 2008). In the socially monogamous prairie vole, being isolated from a pair-bonded partner also increases anxiety-like behavior, but this separation does not affect prairie voles that did not have a strong bond with their mate (Osako et al., 2018). At older ages, the social environment begins to decrease in size and quality, resulting in increased risk for feelings of loneliness. Such social challenges that older adults face include losing a partner, reduced engagement in social activities due to limited mobility or poor health, and being confronted with their own or their partner’s increasing frailty (Dykstra et al., 2005). Additionally, the length of time spent in a relationship and age at which a partner is lost are both positively correlated with stress-related hormone release during isolation in prairie voles (Grippo et al., 2021). Thus, aging is related to increased social experience, and therefore, increased susceptibility to negative outcomes when either the relationship itself, or one’s ability to actively participate in such relationships are lost.

Being close to other individuals can also be stress buffering and protective against the consequences of hardship and adversity. Emotionally, social support is related to more positive moods (Williamson et al., 2019), better mood regulation (Tabatabaei et al., 2018), and lower psychological distress (Lepore et al., 1991; Lee C. Y. S. et al., 2018). Positive social experiences shape our self-perceptions, and thus have a great impact on how we view ourselves, our merit, and our ability to overcome pain or failure. Central to this idea is self-compassion, which has three main components: (1) self-kindness; (2) common humanity, or the view of oneself as part of a broader human experience; and (3) mindfulness of one’s own thoughts and feelings (Neff, 2003). Because our own self-esteem and self-view depends on our perceived feelings of social value and inclusion (Leary and Baumeister, 2000), social support and positive social interactions encourage a more balanced, self-forgiving perspective that may facilitate better emotional well-being. In support, people with high perceived social support have more positive and fewer negative self-perceptions and beliefs about others’ views of themselves (Sarason et al., 1991). Social support is also positively associated with mindfulness, self-compassion, and savoring (the use of thoughts and actions to increase appreciation of positive experiences and emotions), and these three factors significantly mediate the relationship between perceived social support and increased psychological well-being (Wilson et al., 2020). By promoting a more positive view of our ability to navigate hardship, social support may also affect stress-responsivity when experiencing distress. Indeed, social support suppresses cortisol release in humans given a social stress test or experiencing stressful events (Heinrichs et al., 2003; Raymond and Sheppard, 2017), predicts lower cardiovascular reactivity to stress (Howard et al., 2017), and increases pain tolerance (Roberts et al., 2015). Studies in non-human primates also support the stress-buffering role of social affiliation. Introducing a number of aversive stimuli that would typically increase stress responsivity does not do so when animals are presented the stimuli in social groups (Coe et al., 1982; Stanton et al., 1985; Winslow et al., 2003). Stress-buffering can even occur in the absence of physical contact, as exposure to vocalizations of a pair-mate was sufficient to blunt the increase in urinary cortisol of isolated male marmosets (Rukstalis and French, 2005). Studies in rodents have also shown a protective effect of social connectedness against the development of adverse behavioral and physiological consequences. For instance, socially housed rats born to a lineage of stressed mothers (multigenerational ancestral stress) exhibited blunted HPA axis response to chronic stress compared to those that were isolated from peers during stress (Faraji et al., 2017). In the socially monogamous prairie vole (*Microtus ochrogaster*), stress responsiveness is similarly buffered when an animal experiences a stressor in the presence of his or her pair-bonded partner (Smith and Wang, 2014; McNeal et al., 2017; Donovan et al., 2018). Taken together, research from human and animal studies highlight the cognitive, behavioral, and physiological benefits of social affiliation, which implies that removal of such support could have negative repercussions for individual health and well-being.

## Loneliness in Humans Is Associated With Altered Social Behavior

While social affiliation is rewarding and valued, social isolation and ostracization are stressful and aversive. Thus, individuals that perceive being socially isolated, regardless of actual social connectedness, experience feelings of loneliness that are severely distressing. Because loneliness is characterized by the perceived state of isolation, it is unsurprising that lonely individuals have more negative perceptions of everyday interpersonal interactions (Hawkley et al., 2007). This tendency to view social interactions through a negative lens may be driven by previous experience with social relationships. For example, poor-quality social relationships (Mullins and Dugan, 1990; Hawkley et al., 2008) and conflict in current marital or family relationships (Jones D. C., 1992; Burke et al., 2012) are risk factors that predict feelings of loneliness and isolation (Hawkley et al., 2008). As a result, loneliness is also related to stronger expectations of, and motivation to avoid, negative social outcomes and weaker expectations of positive social outcomes (Gable, 2006) as well as increased comorbidity with symptoms of social anxiety (Lieberz et al., 2021a). Because of this fear of social rejection, loneliness is also associated with increased hypervigilance to social threats and, therefore, lonely individuals are more likely to be less trusting and more hostile in uncomfortable social situations (Duck et al., 1994; Anderson and Martin, 1995; Cacioppo and Patrick, 2008; Lieberz et al., 2021b). Despite the tendency to anticipate social rejection, lonely individuals still desire social interaction, behave in ways to promote social connectedness (DeWall and Richman, 2011), and show increased sensitivity to social information (Pickett et al., 2004; Gardner et al., 2005). However, this heightened sensitivity during social interactions, driven by the need to feel socially connected and biased by experience with “failed” relationships, may ultimately prime the expectation of negative outcomes once attempts at social connection are initiated, leading to reduced fulfillment and positive affect following positive social interactions (Lieberz et al., 2021b). Furthermore, this series of events produces anxiety surrounding the social environment, resulting in physiological and health-related consequences that will be summarized in the following section.

## Loneliness in Humans Is Associated With Anxiety and Altered Stress Responsivity

The experience of loneliness is extremely distressing, leading to changes in stress physiology and reactivity in lonely individuals. Stress reactivity is governed by the body’s hypothalamus-pituitary-adrenal (HPA) axis, where stress-related neuropeptides are released from the hypothalamus, causing excretion of “releasing factors” from the pituitary to signal glucocorticoid (cortisol in humans and non-human primates, corticosterone in rodents) release from the adrenal glands. This process is regulated by a negative feedback loop – cortisol released by the adrenal gland will elicit a behavioral response (in concert with the autonomic fight or flight) toward environmental stimuli, but it will also bind to receptors in the brain that turn off the HPA axis which dampens continued cortisol release. Disruptions in both the basal functioning of the HPA axis as well as its ability to appropriately activate and deactivate under stressful conditions have been shown to underlie a variety of psychopathologies (reviewed in Buitelaar, 2013), and similar disruptions can be found in lonely individuals. For instance, lonely people have higher salivary (Steptoe et al., 2004; Doane and Adam, 2010), plasma (Pressman et al., 2005), and excretory (Kiecolt-Glaser et al., 1984) cortisol levels. Trait loneliness is also associated with altered diurnal pattern of cortisol release characterized by lower levels in the morning and afternoon and much higher levels in the evening (Cacioppo et al., 2000). Interestingly, lonely students in this study were just as likely to engage in restorative behaviors (sleep, exercise, spending time with others) as non-lonely students; yet these activities did not seem to blunt stress reactivity as it did in the socially embedded individuals. Basal heart rate and heart rate reactivity after reciting both a social and a non-social speech was also lower in lonely individuals compared to non-lonely individuals, and this suggests blunted autonomic nervous system functioning (Cacioppo et al., 2000). Research in socially deprived chimpanzees has also shown increased HPA and stress reactivity, and resocialization caused a reversal of isolation/deprivation-induced increase in glucocorticoid secretion (Reimers et al., 2007).

## Loneliness in Humans Is Associated With Altered Brain Activation

Loneliness can increase sensitivity to social disconnection, impacting the emotional response to social isolation and the expectations about others. Thus, the perception of isolation may reflect functional changes to brain regions involved in processing social information, regulating and expressing emotion, and assigning reward value to social interactions. In support, the reward-related ventral striatum showed reduced activation in response to viewing images of unfamiliar people in lonely compared to non-lonely individuals (Cacioppo et al., 2009). Furthermore, Inagaki et al. (2016a) found that lonely people had higher activity in the ventral striatum when viewing pictures of familiar people compared to strangers. Thus, it is possible that, due to increased negative expectations and higher anxiety of social outcomes, lonely individuals have a bias against novel social encounters and toward interactions with people they know will respond positively to their attempts for social connection. Moreover, the insula and anterior cingulate cortex are typically activated during presentation of social-related words (i.e. fight, glory) compared to individual words (i.e. talent, pain; Arioli et al., 2021). However, activity in both regions is dampened in lonely individuals during resting state (Morr et al., 2021) while activity in just the insula is blunted in response to viewing pleasant non-social stimuli (Cacioppo et al., 2009) and during participation in a social trust task (Lieberz et al., 2021b). Gray and white matter volume is also lower in the insula and PFC of individuals scoring high on loneliness, which could indicate reduced myelination and/or synapse number that would reduce signal efficacy (Nakagawa et al., 2015; Düzel et al., 2019). While activity is dampened in lonely individuals at rest and during a variety of positive social and non-social stimuli, the insula, ACC, PFC, and ventral striatum are more active in non-lonely individuals that reported feeling excluded during a virtual game (Eisenberger et al., 2003, 2007), and this activity pattern in response to exclusion predicts loneliness scores (Oh, 2020). Thus, it could be that these regions are sensitive to particular information in lonely individuals, i.e., less activation during pleasant stimuli but more activation during aversive or unpleasant stimuli. Furthermore, activation of visual attention regions following presentation of negative social words was greater for lonely compared to non-lonely individuals (Cacioppo S. et al., 2015), suggesting increased sensitivity to negative social stimuli. Altered emotion perception may also be a risk factor for feeling lonely and isolated, as alexithymia (a personality trait characterized by impaired emotional awareness and interpersonal relating) modulates the correlation between insular activity and loneliness (Morr et al., 2021). The insular cortex also integrates sensory input and links these cues with emotional state of both the self and others (Davidson and Irwin, 1999; Ebisch et al., 2011; Zaki et al., 2012; Gu et al., 2013) and is a critical hub for coordinating network switching (Sridharan et al., 2008). Because attention to social cues is heightened in lonely individuals, loneliness may be characterized by disrupted ability to process and relate such social cues to emotional state of social conspecifics, leading to a feeling of unfulfillment after attempts to socially connect (Box 2).

**Box 2. The insular cortex as a neural hub for processing social encounters.**

**FIGURE 1:**
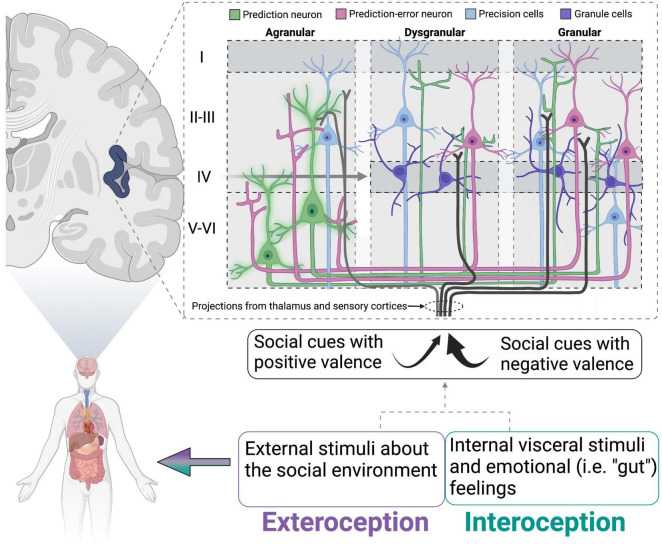
Schematic of proposed insular cortex activity in lonely individuals. Schematic of proposed insular cortex activity in lonely individuals (modeled from Barrett and Simmons, 2015). Prediction neurons (green) are “inflexible” or sending strong prediction signals to dysgranular and granular layers. Simultaneously, social cues/stimuli with negative valence (that match the prediction) are attended to more strongly than those that would generate a prediction error. This would lead to insufficient activation of prediction-error neurons, and therefore no error signal being relayed to prediction neurons to update the simulation. Oxytocin is particularly well situated to modulate the activity of precision neurons and has been hypothesized to do so in a similar model for autism spectrum disorders (reviewed in Quattrocki and Friston, 2014). Adapted from Barrett and Simmons (2015), with permission from Springer Nature.

Recent reviews have postulated a role for the insular cortex in maintaining internal homeostasis through the lens of the inference framework (reviewed in Barrett and Simmons, 2015). This view hypothesizes that the brain forms representations constructed from previous experience while simultaneously using sensory information gathered during current experiences to anticipate a reaction. Thus, perception (based on the integration of past and current experiences) and action are tightly coupled. Extending on theories proposed by Barrett and colleagues who have used predictive processing to describe the development (Atzil et al., 2018) and regulation of social behavior via internal allostatic mechanisms involving the insular cortex (Atzil and Barrett, 2017), we propose that loneliness can be described as a breakdown of these processes. With loneliness being characterized by a strong prediction that attempts at social connection will end poorly, the individual anticipates this response regardless of sensory information currently being gathered that might suggest a different outcome. Furthermore, the insular cortex may play a key role in this process because of its integration of exteroception (perception of external sensory stimuli) and interoception (perception of the internal body) (Kleckner et al., 2017; Koeppel et al., 2020; Lefranc et al., 2020). For example, the agranular insular cortex is thought to contain “prediction neurons” that are ready to respond to an expected outcome (Barrett and Simmons, 2015). These neurons send their predictions in the form of chemical signals to the dysgranular and granular insular layers, altering the firing rate of these neurons in anticipation of sensory information arriving from the thalamus and visceromotor regions in the brain and body (Figure 1). The received sensory signal is compared to the prediction signal and “prediction error” is generated based on how much the dysgranular and granular neurons must change their firing to accommodate the sensory information. The goal of the brain and body is to minimize prediction error and maintain homeostasis, resulting in one of three options: the prediction error signal can be sent back to the agranular layer to update the internal prediction, behavior can be changed to physically modulate the incoming sensory input to match the prediction, or the neurons receiving the sensory signals can bias the influence of particular signals that match the prediction. Appropriate functioning of the insular cortex may be crucial for generating predictions about the social environment. Thus, reduced activation of the insular cortex in lonely individuals at rest or during presentation of social stimuli may reflect an inability to adjust the predicted outcome based on the received sensory stimuli. Furthermore, loneliness is associated with reduced white matter connectivity of the insular cortex to nodes in the attention network (Tian et al., 2014), suggesting that delivery of these signals to the insular cortex could be interrupted or weakened. Some neurons (termed “precision neurons”) in the insular cortex are also responsible for tuning the gain on prediction errors, thereby amplifying some signals and reducing others in an attempt to make sense of the overall input (Shipp et al., 2013; Quattrocki and Friston, 2014). These precision neurons, in particular, could be altered during the loneliness condition, thereby biasing signals that match the prediction (i.e., hypervigilance for social threat) and failing to incorporate others that would update the prediction. Indeed, social exclusion is associated with decreased interoceptive accuracy, which could be explained by altered functionality of the insular cortex. One neurochemical candidate that could be working in the insula to alter appropriate functioning is oxytocin (OT). In rats, OT in the rostral agranular insula is necessary for recognizing affective state of social conspecifics and drives social avoidance of stressed adult rats (Rogers-Carter et al., 2018). Additionally, OT is increased in isolated animals, which could cause over-attention to affective state and ultimately lead to reduced social interaction. Together, this information suggests hyperactivity of attentional networks, but confirmation bias and fear of rejection may prevent such information from being processed and transformed into the desired behavioral output (i.e., social contact).

As an acute response, social isolation should drive an individual to seek out social relationships analogous to the way hunger drives an individual to seek out and consume food. However, the findings synthesized above describe a dampening of motivation-related brain regions. Importantly, recent work has begun exploring the effects of acute social isolation in humans to better understand the shift in brain function and resulting behavioral output from acute to chronic isolation and loneliness. Interestingly, Tomova et al. (2020) found that acute social isolation induces midbrain activation that they likened to craving, as similar responses occurred in people that had fasted. Additionally, social and food craving led to activation of different subregions of the striatum, with social cues evoking responses in the caudate and food cues evoking responses in the nucleus accumbens (Tomova et al., 2020). Finally, they found that the increase in VTA activity and self-reported craving in the socially isolated group was blunted in individuals that scored high on chronic loneliness, suggesting that there is indeed a functional and behavioral shift that occurs when acute social isolation becomes chronic loneliness.

With regards to stress-related brain regions, findings generally indicate increased activation of the amygdala and hippocampus. For example, there is a positive correlation between amygdala volume and social distress score, and this effect was significantly mediated by loneliness (Tian, 2016). Social exclusion also increases activation of the amygdala, and this effect is greater in lonely individuals (Oh, 2020). Increased amygdala activation may be related to attention to socially salient stimuli. For example, socially embedded individuals display stronger coupling of the limbic system with the cortical salience network (which includes the ACC and IC; Bressler and Menon, 2010), while lonely individuals show reduced functional connectivity between these networks (Layden et al., 2017). Oxytocin may be involved in strengthening this connectivity in socially enriched individuals, as OT administration enhances the functional coupling of the amygdala with the cortical salience network during positive social interactions (Rilling et al., 2018). Furthermore, social network size and complexity is positively associated with amygdala volume and functional connectivity of the amygdala to both the affiliation network and the perception network (Bickart et al., 2012). This may reflect an increase in perceptual ability, particularly to affect-laden stimuli, required to foster and maintain a high number of social contacts (for reviews, see Adolphs and Spezio, 2006; Bickart et al., 2014). Because lonely individuals have heightened attenuation to emotional stimuli (Cacioppo et al., 2009), this could explain the increased amygdala activation. The amygdala also responds to threatening stimuli, which may be how lonely individuals perceive social cues based on their hypervigilance and heightened attention during social interaction (Pickett et al., 2004; Gardner et al., 2005). Moreover, reduced connectivity of the amygdala to higher-order cortical regions of the cingulo-opercular and salience networks could indicate an inability of these regions to influence or override the hypervigilance and threat sensitivity that accompanies loneliness. Hippocampal activity is also heightened during a social exclusion task (Eisenberger et al., 2007), and this region hosts a large density of glucocorticoid receptors that act as a negative feedback loop to prevent excess cortisol release during a stressful event (van Haarst et al., 1997). Together, amygdalar and hippocampal sensitivity in lonely persons may reflect their hypervigilance and stress-reactive state.

### Summary of Brain Regions Implicated in Human Loneliness

**FIGURE 2:**
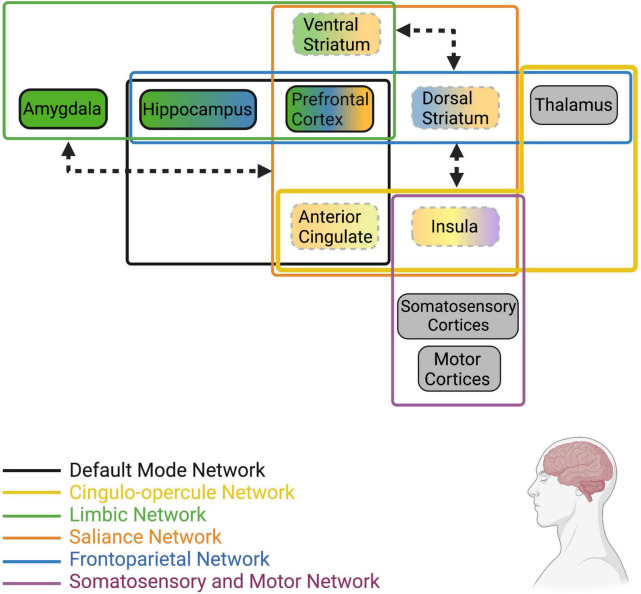
Human brain networks and brain regions within networks that have been implicated in loneliness. Colors represent the network that each region or group of regions belongs to (Note: this is not an extensive list of all known neural networks, only those that are most implicated in human loneliness). Dotted lines represent reduced resting state activity (for individual brain regions) or reduced functional connectivity (arrows between regions/networks) in lonely individuals compared to non-lonely individuals. Bolded lines represent increased resting state activity (for individual brain regions) or increased functional connectivity (arrows between regions/networks) in lonely individuals. Arrowheads touching network boxes denote altered connectivity between networks, while arrowheads touching brain regions denote altered connectivity between regions. Gray boxes represent regions receiving direct stimuli about the environment (i.e., exteroception, interoception, proprioception) and relay such information to other regions defined in these networks; however, information about their resting state activity or connectivity in lonely individuals is unknown at this time. For a recent review of the structural and functional alterations in brain regions and networks associated with loneliness, see Lam et al. (2021).

The hallmark features of loneliness are an increase in desire to engage socially, increased hypervigilance and attention to social stimuli, and disrupted stress-reactivity. As such, loneliness is associated with alterations in resting and stimuli-induced activity in brain regions that are known to regulate these behaviors (Figure 2). For instance, activity and volume of attention and emotion-related regions (i.e., salience and frontoparietal networks) such as the IC, ACC, and PFC are significantly influenced by loneliness, as is activity of limbic regions including the amygdala and hippocampus. Functional connectivity between individual nodes within these networks and among the different networks also appears to be influenced by perceived isolation and loneliness, such that there is generally greater connectivity within regions in the salience and emotion networks and lower connectivity between regions of the salience network to regions of the limbic network. Taken together, the outlined activation and connectivity patterns within the brain of lonely individuals suggests reduced reaction to positive social stimuli, heightened reaction to negative social stimuli, reduced ability of the salience network to override limbic hyperactivity/hypervigilance, and elevated alertness/attention.

## Loneliness Modeling in Animals

### Animal Behavior Mimics the Human Loneliness Condition

Animal research is a valuable aid for understanding the mechanisms involved during the experience of social isolation; however, there will inevitably be gaps due to the perceptive component to human loneliness that may not be possible to accurately model using animals. So far, we know that social isolation causes a similar behavioral phenotype to human loneliness, characterized by increased desire for social contact (Panksepp and Beatty, 1980; Ikemoto and Panksepp, 1992; Panksepp and Burgdorf, 2000) followed by a deficiency in social interaction once it’s been obtained (Zelikowsky et al., 2018; de Moura Oliveira et al., 2019; Kaneda et al., 2021), increased stress- and depression-related behaviors (Parker and Morinan, 1986; Hellemans et al., 2004; Grippo et al., 2007b; Yorgason et al., 2013; Lander et al., 2017; Zorzo et al., 2019), cognitive deficits such as poor spatial learning and memory (Hellemans et al., 2004; Lander et al., 2017; Zorzo et al., 2019) and impaired novel object discrimination (Bianchi et al., 2006), increased threat responsiveness (Zelikowsky et al., 2018), and avoidance of (and hyper-activity when exposed to) new experiences/stimuli (Parker and Morinan, 1986; Gentsch et al., 1988; Varty et al., 2000). This behavioral phenotype is driven by alterations in a host of neurochemicals in a variety of brain sites, detailed below.

### Alterations in Neurotransmitter and Neuropeptide Systems

Neurobiologically, the display of social behaviors in non-human animals is governed by similar brain regions and neurochemicals as they are in humans. For instance, DA in the ventral striatum, specifically the NAc, is the “end point” that drives motivated behaviors including social affiliation (reviewed in Floresco, 2015). The NAc is also modulated by OT, endogenous opioids (i.e., endorphins), and stress-related neuropeptides, the integration of which adjusts the salience of social stimuli depending on context and physiological state (Mannella et al., 2013). Using rodents to assess the effects of social isolation on the brain and behavior, interactions between DA, OT, stress neuropeptides, and endorphins both widely and in the NAc specifically have been identified and implicated in the behavioral adaptations that occur during isolation. It should be noted that the effects of social isolation on rodent social and emotional behavior and neurophysiology do vary based on the age at which the isolation occurs and the length of time that isolation is experienced. However, this review will mainly focus on isolation during adulthood unless otherwise specified. For a detailed review of the effects of social isolation during adolescence, see Walker et al. (2019).

#### Oxytocin

Because of its role in promoting affiliation, it is unsurprising that OT is protective against the negative behavioral and physiological consequences of social isolation. For instance, isolation-induced increased basal heart rate and reduced vagal regulation of the heart was reversed by systemic OT administration (Grippo et al., 2009). OT treatment also prevented isolation-induced cellular aging, characterized by oxidative damage and telomere degradation (Stevenson et al., 2019). Interestingly, social isolation is associated with an increase in the density of OT cells in the paraventricular nucleus of the hypothalamus (PVN), and an increase in the number of these cells that are activated via co-expression of the immediate early gene, cFos (Grippo et al., 2007a). Similarly, male and female hamsters that were socially isolated at weaning had higher OT mRNA in the PVN and lower oxytocin receptor (OTR) binding in the NAc in adulthood (de Moura Oliveira et al., 2019). Stress-induced serum OT was also heightened in animals that have been socially isolated (Grippo et al., 2007a). On the surface, these findings could be considered contradictory to the traditional anxiolytic view of OT (i.e., high OT = low anxiety). However, this heightening of endogenous OT activity during social isolation could indicate abnormal homeostatic processes, as acutely, OT is released from the PVN to facilitate appropriate behavioral responses to unpredictable threats (Dabrowska et al., 2011; Knobloch et al., 2012). Building on the concept of abnormal homeostatic mechanisms, Musardo et al. (2021) found that acute social isolation resulted in increased social interaction and a disruption in social habituation and social novelty preference. While these behaviors could be viewed as negative outcomes, it could be the result of brain mechanisms promoting social contact regardless of familiarity or length of contact (i.e., fewer “restrictions” on who to interact with as long as social contact is achieved). Importantly, inhibiting OT neurons in the PVN abolished the above behavioral effects, suggesting that increased OT release during acute isolation may be at least partially responsible for increased social-seeking behavior aimed at achieving social homeostasis. Oxytocin in the insular cortex, a region known to be less active in lonely humans, is also necessary for recognizing social affective stimuli. Adult male rats have a natural tendency to prefer interacting with stressed juvenile rats and avoid stressed adults, and blocking OTRs in the rostral agranular IC (RAIC) reduces interaction with stressed juveniles and increases interaction with stressed adults (Rogers-Carter et al., 2018). Moreover, OT injected into the RAIC further reduced social interaction with a stressed adult. Thus, increased OT release into the RAIC, presumed via upregulated PVN OT release during these conditions, could contribute to social avoidance.

Oxytocin also influences the HPA axis differently depending on the stress state of the animal. For example, blocking OT receptors in the PVN under basal conditions resulted in enhanced secretion of adrenocorticotrophic hormone (ACTH) from the pituitary gland, while under stress conditions OTR blockade reduced ACTH secretion (Neumann et al., 2000a,b). Additionally, OT administration prior to an elevated platform stress inhibits stress-induced CRH release via activation of GABA neurons, while post-stress OT administration has no effect on CRH release (Smith et al., 2016). These findings suggest that OT may suppress HPA axis function normally, but during stress experience facilitates HPA activity. Furthermore, these divergent roles of OT may involve differences in OT neuron population, receptor distribution, or receptor sensitivity under various conditions. OTRs are widely distributed throughout the brain and once bound to OT, can have varying effects on social behavior depending on the brain region being influenced. For instance, activating OT neurons in the bed nucleus of the stria terminalis (BNST) causes increased vigilance behavior in socially defeated female California mice (*Peromyscus californicus*) (Duque-Wilckens et al., 2018), while activating OTRs in the NAc promotes pair bonding in prairie voles. OTR distribution and sensitivity during isolation conditions could underly the ability of OT to promote or inhibit social interaction. Therefore, a disruption in the balance of OTRs and OT release in isolated animals may contribute to the altered social and stress-related behavior observed during isolation. To date, no studies exist that directly manipulate OTR activation in specific brain sites under isolation conditions; however, OTR expression does indeed vary by brain region in response to social isolation. OTR mRNA is increased in BNST (Perkins et al., 2019) following chronic social isolation, while OTR binding was lower in the DR of isolated female Syrian hamsters (Ross et al., 2019) and in the CeA of isolated male rats (Han et al., 2018). Thus, manipulating OTR activity in particular brain regions during social isolation would likely produce varying effects on behavior.

To add another layer of complexity, other neurotransmitters and neuropeptides interact with the OTergic system to modulate its regulation of social and stress-related behavior, and in turn, OT can do the same to those other systems. For instance, serotonin (5-HT) directly influences OT release via 5-HT1A and 2A receptors in the PVN, and this interaction has been shown to affect social behavior (Edwards et al., 2018). Similarly, DA modulates OT release and influences socio-sexual behavior via direct projections of DA fibers from the incertohypothalamic system to the mPOA, SON, and PVN (Buijs et al., 1984; Decavel et al., 1987; Holstege et al., 1996; Baskerville et al., 2009). The influence of OT on other systems appears to be widespread, as it not only interacts with 5-HT and DA, but also directly influences CRH and opioid activity. To list a few examples: OTRs expressed on serotonin (5-HT) terminals in the NAc are critical for social motivation (Dölen et al., 2013), OTR activation in the VTA is necessary for social reward in male and female Syrian hamsters (Borland et al., 2019), OT modulates HPA activity and CRH release via interaction with GABA-A receptors in the PVN (Smith et al., 2016), and OT binds to Mu opioid receptors as a positive allosteric modulator and directly influences anxiety-like behavior associated with chronic pain (Meguro et al., 2018; Miyano et al., 2021). Finally, the social salience hypothesis of OT proposes that OT regulates the salience of social stimuli and increases sensitivity to social cues depending on context and individual factors (reviewed in Shamay-Tsoory and Abu-Akel, 2016). The mechanism underlying this is also thought to involve interaction with the DArgic system since it governs the processing of aversive and rewarding events. Thus, OT interacting with other neurotransmitters and peptides expands its potential involvement in social isolation and the resulting stress-related behavior in a complex manner and further emphasizes the need for continued research on the nuanced neural mechanisms underlying these behaviors.

#### Dopamine

As stated above, DA is critically involved in the ability to receive, assign valence, and respond appropriately to a variety of rewarding and aversive stimuli. OT may also boost DAs role in social attention by increasing the valence of social stimuli, as both DA and OT are required for pair bond formation in prairie voles (Liu and Wang, 2003; Romero-Fernandez et al., 2013), and OT further activates the DA system during times in which social affiliation is increased (such as when maternal rats lick their pups; Shahrokh et al., 2010). In addition to its general role in social motivation, DA signaling has been shown to affect social-seeking behavior under isolation conditions. In fact, Matthews et al. (2016) propose that DA neurons in the midbrain dorsal raphe nucleus encode the experience of loneliness. They elegantly showed that activating these DA neurons in a social context promotes social interaction, while activating these neurons in the absence of a social conspecific is aversive. These DA neurons from the dorsal raphe almost exclusively innervate the amygdala and extended amygdala (i.e., central amygdala and bed nucleus of the stria terminalis; Hasue and Shammah-Lagnado, 2002) and regulate different behaviors depending on their downstream target. For instance, DRN DA neurons projecting to the CeA promote sociality, while those projecting to the posterior basolateral amygdala (BLP) regulate negative affective state (Tye et al., 2021). DA DRN neurons are involved in the perception of salient stimuli promoting vigilance and arousal (Cho et al., 2017). Because isolation increases glutamatergic tone on DA DRN neurons, the basal activity of these neurons in socially isolated animals creates an aversive experience that causes seeking of social contact to ameliorate the unpleasant state of being isolated. Another distinct DA circuit that is influenced by social isolation is the DA afferent projections from the VTA to corticostriatal regions including the prefrontal cortex and nucleus accumbens, though much of this work has been done in adolescent rodents. Specifically, DA neurons in the VTA are more sensitive in socially isolated rats, as stimulating them causes greater DA release in the NAc compared to group-housed rats (Karkhanis et al., 2018). This increased DA release in socially isolated rats can be evoked by both rewarding and aversive stimuli (Lapiz et al., 2003; Yorgason et al., 2013, 2016; Karkhanis et al., 2016). Basal extracellular DA level is not affected by social isolation (Jones G. H., 1992; Karkhanis et al., 2014), though some studies report an increase in DA turnover in the NAc (Hall et al., 1998). Postpartum rats that have had their pups removed and were socially isolated for 3 weeks showed reduced basal activity of DA neurons in the VTA accompanied by increased passive coping and reduced social motivation (Rincón-Cortés and Grace, 2021). Interestingly, when dams were housed with another postpartum rat that had her litter removed as well, passive coping behavior was partially ameliorated and VTA DA activity was restored.

The balance of basal and stimuli-induced DA release is critical for the appropriate processing of salient stimuli. For example, maternal rats experience a reduction in baseline DA and increased sensitivity and response of DA neurons to pup-related cues (Robinson et al., 2011) that allows them to respond with the appropriate behavior quickly. In the case of social isolation, it appears that the ability to discern between various stimuli may be impaired since high DA responsiveness occurs regardless of the stimulus. Interestingly, depleting DA in the NAc reverses the increase in pre-pulse inhibition response of socially isolated rats (Powell et al., 2003). This suggests that the signal to noise ratio might be low following social isolation, creating an inability to discern between salient stimuli and reducing the noise allows for more appropriate gating of behavioral responses.

Acutely and via the DArgic system, social isolation may drive social-seeking behavior as a mechanism to restore social homeostasis (reviewed in Matthews and Tye, 2019). However, as the isolation becomes more chronic and/or more distressing, the experience of social contact becomes more stressful due to increased hypervigilance and over-attention to social cues, and also less rewarding. Still, there is evidence that these long-term, isolation-induced alterations in DA signaling can be reversed by successful social reintroduction, suggesting that this shift from adaptive mechanism to maladaptive behavioral inflexibility can be undone. For example, non-human primates that were socially isolated for up to one and a half years were then reintroduced into social groups consisting of four animals. Repeated positron emission tomography (PET) scanning showed an increase in D2 receptors in the basal ganglia after as little as 3 months of social re-housing (Morgan et al., 2002). The neurobiological underpinnings governing the shift from homeostatic resolution to chronic reactivity and hypervigilance have not been clearly identified. Efforts to investigate this phenomenon could prove to be invaluable for developing therapeutic treatments for loneliness and isolation in humans.

#### Opioids

Endogenous opioids consist of three main groups: endorphins, enkephalins, and dynorphin. These peptides vary in both structure and binding affinity to the three main opioid receptors. Endorphin primarily binds to the mu opioid receptor (μ; MOR) and delta opioid receptor (δ; DOR), enkephalins have an affinity for DOR, and dynorphin binds primarily to kappa opioid receptor (κ; KOR). The opioid system is closely linked to the DArgic system as it is highly involved in motivated behavior. Additionally, the opioid system is a key regulator of pain sensation via both mu opioid and kappa opioid receptors. The sensation of pain can be viewed as an alert signal that tells the organism when something is not right. In the case of physical pain, humans and non-human animals respond quickly with a reflexive movement to alleviate the pain accompanied by release of endogenous opioids that ease the pain response (Mayer and Saper, 2000). Since pain is aversive, it is possible that the displeasure of being socially isolated could be processed by pain-sensing brain regions and include the involvement of endogenous opioids. In fact, “emotional pain” is an increasingly more common term used to describe feelings of social exclusion in humans (Mee et al., 2006; Eisenberger, 2008). In humans, involvement in intimate relationships elevates pain thresholds (Younger et al., 2010; Che et al., 2018), while in prairie voles, physical pain sensitivity is positively correlated with the anxiety-like symptoms produced by social isolation via partner loss (Osako et al., 2018). Because of its role in both reward and pain signaling, the opioid system is uniquely situated to modulate the interception of these states during social isolation. Indeed, opioids are broadly involved in social behavior, as opioid receptor agonists have been shown to reduce time spent in close proximity to social conspecifics (Herman and Panksepp, 1978; Panksepp et al., 1979), while opioid antagonists increase attempts to engage socially through allogrooming (Fabre-Nys et al., 1982; Martel et al., 1995). These behavioral responses are thought to be driven by interaction of opioids with the reward system, as activating opioid receptors would provide positive reward state and thus suppress the need to seek out pleasurable experiences via social contact. Similarly, blocking these receptors would produce an aversive experience and drive behaviors to ameliorate those feelings by engaging in affiliative behavior. Opioid administration also reduces the distress experienced by separation/isolation, as evidenced by reduced vocalizations of rat pups separated from their mother (Panksepp et al., 1978), and social affiliation increases endogenous opioid release (Keverne et al., 1989). Social isolation in rodents is also accompanied by reduced mRNA expression of mu and kappa opioid receptors in the hippocampus and amygdala, while systemically injecting a DOR agonist reverses isolation-induced anxiety-like behavior (Haj-Mirzaian et al., 2019). In humans, blocking opioid receptors reduces feelings of social connectedness, reduces perceptions of emotional and physical warmth, and reduces activity in the ventral striatum (Inagaki et al., 2015, 2016b,^2020^). Variation in human attachment is also associated with the opioid system, as humans with more avoidant attachment styles have lower mu opioid receptor availability, particularly in the anterior cingulate and insular cortices, while secure attachment was associated with higher MOR availability (Nummenmaa et al., 2015; Turtonen et al., 2021).

While mu opioid receptors have gained the most attention regarding the positive and rewarding aspects of social behavior, kappa opioid receptors (KORs) may mediate the aversive experience of stress, particularly via coordination with DA circuits (Margolis and Karkhanis, 2019). Forced swimming and foot shocks both produce aversive responses that are reversed by blocking KORs or deleting the KOR gene (i.e., KOR knockout mice; Land et al., 2008). This study also localized KORs involved in stress responsiveness to the BLA, NAc, DR, and hippocampus by showing their activity both during stress and during administration of corticotrophin releasing hormone (CRH). KOR blockade or deletion from DA transporter (DAT)-expressing cells in the NAc also prevents defeat-induced anhedonia when the social defeat is acute, but has no effect when defeat is chronic (Donahue et al., 2015), suggesting a shift in the functionality of KORs the longer a stressor is experienced. Thus, the opioid system may integrate motivation and stress-related circuits to assess threat, social stimuli, and motivational state to initiate a behavioral response. The opioid system may further modulate the connection between stress and social affiliation by acting directly on the OT and CRH systems. For instance, administering an exogenous opioid receptor agonist (i.e., morphine) enhances HPA axis responses to acute stress, while blocking opioid receptors reduces stress responsiveness (Buckingham and Cooper, 1986; Buller et al., 2005). Additionally, opioid receptor activation via morphine (exogenous), met-enkephalin (endogenous), and leu-enkephalin (endogenous) all directly stimulate CRH secretion from the hypothalamus (Buckingham and Cooper, 1984). Interestingly, the influence of endogenous opioid on HPA activity may change depending on physiological state. In support, blocking opioid receptors in pregnant rats leads to an increase in stress-induced CRH and OT release, indicating a tonic inhibitory effect of opioids during pregnancy (Brunton et al., 2005).

Comparing opioid involvement in social behavior to its involvement in drug addiction, it has been implicated that, acutely, social isolation may mimic a withdrawal state characterized by reduced endorphin release (via reduced social affiliation) in order to drive motivation for seeking out social contact (reviewed in Machin and Dunbar, 2011; Burkett and Young, 2012). Additionally, endorphin withdrawal coupled with a stress-induced increase in the dysphoria-inducing PDYN/KOR system could compound to create a strong desire to achieve social homeostasis. However, as social isolation becomes chronic, leading to alterations in physiological state characterized by high basal glucocorticoid secretion and exaggerated HPA activity, it’s possible that constant hypervigilance and stress-reactivity overrides the ability of the opioid system to respond during subsequent social interactions.

#### Neuropeptides (Corticotrophic Releasing Hormone and Tachykinin Peptides)

As has been outlined in this review, it is stressful to be alone and isolated from kin and peers. Both chronic and acute social isolation are characterized by altered responsiveness of the HPA axis, and thus a disruption in the production and release of the neuropeptides that regulate it. Acute (7 h) isolation from a partner significantly increases cortisol in marmosets, while blocking CRH-1 receptors during separation blunted this effect and increased sociosexual behavior without affecting alarm calls (French et al., 2007). Thus, CRH receptor activation appears to be involved in HPA responsiveness and anxiety-like behavior during acute isolation. In prairie voles, a single isolation event or repeated isolation, but not chronic isolation, result in higher expression of CRH mRNA in the hypothalamus and hippocampus of both males and females (Pournajafi-Nazarloo et al., 2009, 2011). All three isolation conditions result in reduced mRNA expression of CRH-R2 receptors in the hypothalamus, while chronic isolation was also associated with higher expression of this receptor subtype in the hippocampus (Pournajafi-Nazarloo et al., 2009) and reduced expression in cardiovascular tissue (Pournajafi-Nazarloo et al., 2007). Interestingly, one day of re-pairing with a sibling does not reverse the altered mRNA expression in repeated or chronically isolated animals (Pournajafi-Nazarloo et al., 2007, 2009). Additional studies have found that CRH binding protein (CRH-BP) is significantly influenced by social isolation. CRH-BP was originally thought to sequester CRH by binding to it and making it less available to bind to its receptors. However, more recent studies suggest that it may actually enhance CRH signaling through trafficking of CRH receptors to the cell surface (Ketchesin et al., 2017). Crhbp mRNA expression is significantly reduced following 24 h of isolation (Leser and Wagner, 2015; Lavenda-Grosberg, 2019), while the protein product of this gene is significantly more abundant in acutely isolated animals (Lavenda-Grosberg, 2019).

Although there are currently no studies that have directly manipulated the CRH system in socially isolated animals, this system has been the target of other studies utilizing various social stress paradigms. CRH-R1 receptor blockade reduces defeat-induced immobility in rats (Wood et al., 2010), while non-selective CRH blockade injected directly into the DR of Syrian hamsters reduced the acquisition and expression of conditioned defeat by reducing submissive and defensive behavior (Cooper and Huhman, 2007). Since chronic social defeat and chronic social isolation both upregulate the HPA system, including an increase in CRH-related gene and protein expression (Marini et al., 2006; Pournajafi-Nazarloo et al., 2009), it could be expected that directly manipulating the CRH system in isolated animals would impact stress-related behaviors. CRH may also affect social behavior during isolation, as central injection of CRH reduces the number of social interactions in rats (Dunn and File, 1987) and mice (Mele et al., 1987). Site-specifically, infusing CRH or urocortin-1 into the basolateral amygdala reduces social interactions (Rainnie et al., 2004; Spiga et al., 2006), while infusing urocortin-1 into the BSNT induces anxiety-like behavior during a social interaction test (Lee et al., 2008). Thus, the general effect of central CRH release, at least during a stress-naïve state, appears to be anti-social. Importantly, while increased CRH is typically indicative of an anxiogenic state, pair bonding in male prairie voles is accompanied by an increase in CRH mRNA in the BNST (Bosch et al., 2009) and CRH facilitates social bonding in males (DeVries et al., 1996, 2002). In females, however, glucocorticoid injection prevents pair bond formation while adrenalectomy facilitates it (DeVries et al., 1995). This sex difference could play a role during social isolation as well, because female prairie voles that were isolated for 6 weeks, a time point that has been shown to increase CRH immunoreactivity in the hypothalamus (Grippo et al., 2007a) showed increased affiliative behavior (Lieberwirth et al., 2012). Furthermore, CRH infused directly into the insular cortex suppresses presynaptic GABAergic neurotransmission, leading to greater excitatory synaptic efficacy, and increases social investigation in male rats but not female rats. The insular cortex is also responsible for detecting stress state of other animals, as inactivating the IC prevents the preference that male rats have for interacting with stressed juveniles and their avoidance of stressed adults (Rogers-Carter et al., 2018). CRH-R1 receptors in the IC appear to be necessary for this social decision-making process, but only in male rats (Rieger et al., 2021). These findings describe a complex role for CRH activity in regulating social and affiliative behavior that varies based on sex and stress experience.

While CRH has received much attention due to its widely known role in stress and anxiety-related behaviors, other work has shown that social isolation may affect social behavior via tachykinin peptides. Specifically, tachykinin 2 (Tac2) mRNA and neurokinin B (NkB) immunostaining are upregulated in the dorsal BNST, central amygdala (CeA), dorsomedial hypothalamus (DMH), and ACC following social isolation (Zelikowsky et al., 2018). Additionally, blocking Nk3 receptors, which show the highest affinity for NkB, reverses social isolation-induced aggression and heightened threat response. Tac2 knockdown in the DMH attenuates isolation-induced aggression, while Tac2 knockdown in the CeA attenuates threat and fear responsiveness, showing that Tac2 expression in specific brain regions differentially mediates isolation-induced social and stress-related behaviors.

### Summary of Neurobiology Underlying Social Isolation

**FIGURE 3:**
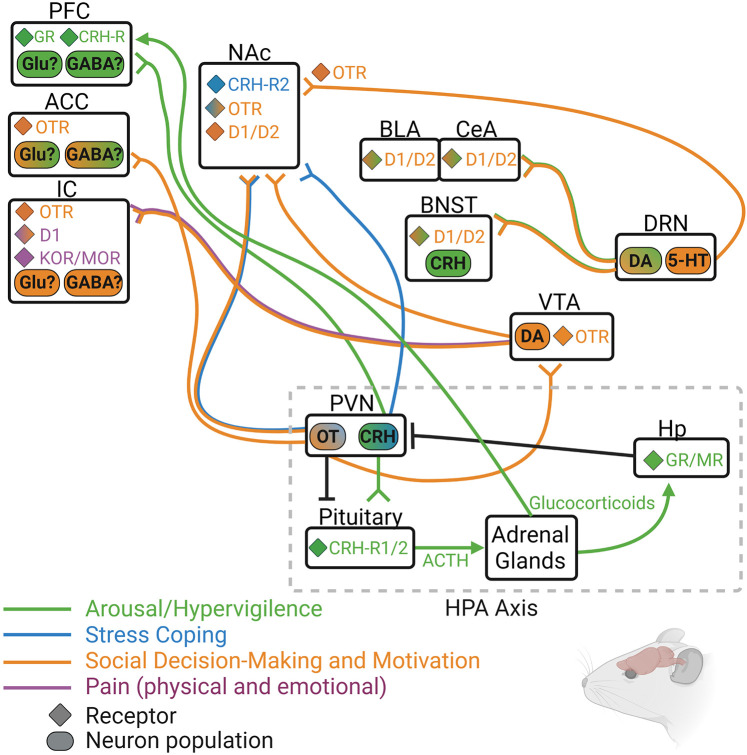
Rodent model depicting some of the known neurochemical circuits that regulate hypervigilance (green), social motivation (orange), passive coping (blue), and pain (purple), with particular focus on the four main neurochemical systems discussed in this review: oxytocin (OT/OTR), dopamine (DA/D1/D2), endogenous opioids (DOR/KOR/MOR), and corticotrophic releasing hormone (CRH/CRH-R1/2). 5-HT, serotonin; ACC, anterior cingulate cortex; ACTH, adrenocorticotrophic hormone; BLA, basolateral amygdala; BNST, bed nucleus of the stria terminalis; CeA, central amygdala; DRN, dorsal raphe nucleus; GR, glucocorticoid receptor; Hp, hippocampus; IC, insular cortex; MR, mineralocorticoid receptor; NAc, nucleus accumbens; PFC, prefrontal cortex; PVN, paraventricular nucleus of the hypothalamus; VTA, ventral tegmental area.

Animal models of social isolation reveal a number of alterations in neurobiological systems that regulate social behavior, motivation, and stress reactivity (see Figure 3 for circuit summary). Although this review focused only on OT, DA, opioids, and CRH, it is known that other neurotransmitter and neuropeptide systems are involved in the physiological and behavioral effects of social isolation in both humans and non-human animals. Still, it is likely that at least these four systems influence isolation-induced behavior in humans in a similar manner to their influence in animals. For instance, OT has been consistently shown to affect social and affection-related behavior in humans, even in lonely or excluded individuals (Lucht et al., 2009; Norman et al., 2011). DA and endogenous opioids are also known to underly the pleasurable experiences of social touch and other rewarding aspects of social connection (Inagaki et al., 2015, 2016b,^2020^), and activity and connectivity of DA- and opioid-rich brain regions such as the ventral striatum, insula, and anterior cingulate cortex is also disrupted in lonely individuals (Cacioppo et al., 2009; Nakagawa et al., 2015; Morr et al., 2021). Finally, HPA responsivity is disrupted in lonely individuals, and downstream targets of HPA activity including the amygdala and hippocampus show altered activation in lonely people (Tian, 2016).

## Areas of Mutual Interest for Human and Animal Researchers Studying Loneliness and Isolation

Several reviews have been published identifying the neurobiological correlates for loneliness in humans and the behavioral and neurophysiological effects associated with social isolation in rodent models (Cacioppo J. T. et al., 2015; Mumtaz et al., 2018; Tomova et al., 2021). As mentioned previously, it is difficult to directly compare the two literatures because of the largely subjective nature of loneliness in humans. However, here we will highlight some similarities in the neural correlates and brain regions affected by both experiences and will draw from the more detailed knowledge of the neurochemical underpinnings of isolation garnered from the animal research to make hypotheses about their contribution in the human loneliness condition.

One important aspect of human loneliness that is conserved in animal models using social isolation is the high-stress state that is produced as a result of being either subjectively or objectively isolated. In humans, loneliness is associated with increased social anxiety (Lieberz et al., 2021a), heightened attention to social cues (Pickett et al., 2004; Gardner et al., 2005) and increased amygdala activity and gray matter volume (Tian, 2016). In adult prairie voles, chronic social isolation reduced the total number and the overall length of dendritic branches while increasing the spine density in interneurons within the BLA (Hylin et al., 2022). These results suggest a possible reduction in the number of neuronal signals received via reduced dendritic matter and a simultaneous increase in synaptic efficacy of the signals that are received via increased number of synapses. This could indicate a biasing or “tuning in” of particular BLA neurons to specific signals being received from certain brain regions. One projection that could be biased is that of DA neurons coming from the DR, as they are especially active during isolation. In fact, DR DA neurons almost exclusively project to limbic regions, and those that terminate in the amygdala drive social behavior and negative affective state in isolated rodents. OT may also be involved, as plasma levels are increased during chronic social isolation in rodents (Hylin et al., 2022), and in humans OT modulates attention to both positively and negatively valenced social cues (Domes et al., 2007; Gamer et al., 2010). OT neurons also project to dopaminergic neurons in both the VTA (Hung et al., 2017) and, although to a lesser extent, the DR (Otero-García et al., 2016; Fujisaki et al., 2020). Silencing PVN OT neurons in juvenile rats that have been socially isolated for 1 week restores social novelty preference, indicating that over-activation of these neurons during isolation may drive input to DA neurons to increase their activity and alter social behavior (Musardo et al., 2021).

In lonely humans, reward-related brain regions show reduced activation, which may seem counter to the above findings in rodents indicating heightened activity of DAergic regions; however, acute isolation in humans does increase VTA activity in response to social cues, although when loneliness was factored into these analyses the increase in VTA responsiveness to social cues was blunted in lonely individuals (Tomova et al., 2020). Thus, it is unclear whether DA activity remains heightened as acute social isolation becomes chronic or as objective isolation becomes subjective (i.e., loneliness). Excessive DA release in the dorsal striatum does reduce social behavior and social exploration in adult non-isolated mice (Lee Y. et al., 2018). Additionally, continuous over-activation of DAergic system over a long period of time would likely cause alterations in receptor expression or responsiveness, which could affect the strength and efficiency of the signal being received by downstream DAergic target regions. To this point, some downstream targets of DA are more active in lonely humans (amygdala and hippocampus) while others are less active (ACC, insula). If overall DA activity is heightened during loneliness and social isolation, this could simultaneously increase the desire to interact socially while promoting increased attention and hypervigilance during said social encounters, which may alter the social experience to provide less reward value. Furthermore, while human loneliness is associated with reduced activation of reward regions in the brain both at resting state and during presentation of social stimuli, activity in these regions is increased during presentation of familiar faces (Inagaki et al., 2016a). An interesting animal correlate to this behavior is one that researchers typically interpret as a social learning deficit, characterized by the inability of a rodent that has been socially isolated to spend more time with a novel animal over an animal they have already interacted with (Shahar-Gold et al., 2013; Lavenda-Grosberg et al., 2021). One could alternatively interpret this behavior as a familiarity preference rather than a lack of novelty preference. If an individual is anxious about social encounters, they may be more likely to interact with the social stimulus that they already know rather than a novel one that they have not encountered before. Alternatively, the social homeostasis model (reviewed in Lee et al., 2021) postulates that after continued attempts to engage in social behavior during acute isolation, animals will eventually switch from an active coping strategy to a passive one, leading to an adjustment in homeostatic set-point for social contact. In other words, the neural circuits that promote social behavior during acute isolation (driving an animal to get back to its social set point by restoring social connections) are the same ones that promote antisocial behavior during chronic social isolation (driving an animal to maintain it’s new social set-point by avoiding social conspecifics). Thus, reduced social novelty preference during chronic isolation may instead be the result of homeostatic mechanisms geared toward maintaining the new social set-point that is now characterized by a social deficit.

In mice, one brain region that drives novelty preference is the interpeduncular nucleus (IPN), as photoactivating this region reduces exploration of a novel social stimulus, mimicking familiarity, while reducing activation of the IPN during social conditioned place preference increases the rewarding properties of a familiar social encounter (Molas et al., 2017). As a novel social stimulus becomes familiar, activity within the IPN increases via cholinergic-glutamatergic projections from the medial habenula (mHb) to the IPN, whereas during an encounter with a novel stimulus, VTA-IPN projections activate D1 receptors to increase the salience of the novel stimulus to promote exploration. Post-weaning social isolation blunts activation of the Hb in response to a novel social encounter (Ahern et al., 2016); thus, reduced Hb activity resulting from isolation may prevent the encoding of a novel stimulus as familiar in order to bias interaction with familiar social conspecifics. The Hb and IPN have been understudied in lonely humans; however, novelty is also detected by higher order brain regions within the cognitive control networks, many of which are blunted in activity in lonely individuals (Cacioppo et al., 2009; Inagaki et al., 2015; Wong et al., 2016). These cognitive control networks, including the frontoparietal and cingulo-opercule networks, are also involved in conflict-detection, goal-oriented behavior, and behavioral flexibility, and disruptions in the ability to perform such tasks could underly some of the behavioral phenotype featured during loneliness.

Finally, the human and animal literatures could benefit from a more cohesive research trajectory. Although methodologically difficult, future research in humans could attempt to identify neurotransmitter and/or neuropeptide systems that are affected by perceived social isolation, either by measuring plasma or CSF concentrations of these neurochemicals or using PET ligands. Meanwhile, animal studies can begin further characterizing the neural circuits that regulate the behavioral phenotypes resulting from various lengths of isolation, particularly related to regions that show altered activity or connectivity in lonely humans. Additionally, a more specific time course could be identified characterizing the shift from normal, sporadic feelings of loneliness (and isolation) to pathological and chronic loneliness. Lee et al. (2021) make the point that the behavioral shift that occurs between acute and chronic isolation may be mechanistically linked to one or more variables other than time, including effort expended or number of rejected attempts. Assessing more specific factors that contribute to or influence feelings of loneliness (or the behavioral correlates in animals) is an area that would be interesting for both human and animal researchers to explore.

## Social Loss as a Unique Form of Social Isolation

Losing a relationship happens naturally in social species. A relationship may dissolve due to conflict, when one individual leaves to join a new social group, or even from death of a loved one. We know that the response of an individual to social loss depends on the strength of the relationship and the type of relationship (Smigelsky et al., 2019; Sekowski and Prigerson, 2021); however, the mechanisms involved in the behavioral and physiological response to loss is poorly understood. Additionally, examining how the brain adapts to loss and overcomes the negative consequences of loss to allow an individual to form new relationships could provide valuable insights for recovery during grief and loss in humans.

### Grief and Bereavement in Humans

Each year in the US, approximately 8 million individuals suffer from the loss of a friend or loved one, leaving over 800,000 new widows and widowers who are at increased risk of mortality during the first 6 months following their loss (Rees and Lutkins, 1967; Hensley et al., 2009). Similar to social isolation, social loss is distressing and leads to increased stress, anxiety, and feelings of loneliness (Zisook and Shear, 2009; Fried et al., 2015). However, one key difference is that increased social connectedness does not reverse the feelings of loneliness and isolation in bereaving individuals. In support, married women with low perceived social support report feeling lonelier than those with high perceived social support, while widowed women report high emotional and social loneliness regardless of their perceived social support (Stroebe et al., 1996). Additionally, while loneliness decreases over time in bereaving individuals, so does social support, suggesting that the two are not inversely related (Utz et al., 2014). Bereavement and social isolation are also characterized by alterations in brain activity in the same brain regions, such as the insula and anterior cingulate cortex (Gündel et al., 2003; O’Connor et al., 2007). However, lonely individuals display reduced activity in these regions both at baseline and in response to presentation of social-related cues (Nakagawa et al., 2015; Morr et al., 2021), while those experiencing loss have significantly increased activity in the insula and anterior cingulate cortex following presentation of a picture of the deceased individual (Gündel et al., 2003; O’Connor et al., 2007). Interestingly, lonely individuals that view photos of a familiar social conspecific do display higher activation of the insula compared to when they view photos of a stranger (Inagaki et al., 2016a). These results suggest that the insula may be more sensitive to familiar social cues than to unfamiliar social cues during loneliness. The insula is involved in emotional processing and empathy, which both may be increased when viewing pictures of someone familiar and especially in someone who is craving social interaction with a particular person. Interestingly, increased activity of the insula in bereaving individuals is reduced as time since loss increases, assuming that the individual is coping well. However, one distinct brain region that remains highly activated in persons having trouble coping with loss (termed complicated grief) is the ventral striatum (i.e., NAc; O’Connor et al., 2008). O’Connor et al. (2008) propose that over time, and in individuals with non-complicated grief, the NAc becomes less activated by reminders of the lost loved one. Meanwhile, those experiencing complicated grief continue yearning for the deceased individual through increased responsiveness of the NAc. The NAc receives connections from both the insula and anterior cingulate cortex. Activation of the insula during reminders of the deceased loved one could cause excitation of the NAc, leading to a yearning response. Another study found that complicated grief was associated with reduced rostral anterior cingulate cortex activation compared to the non-complicated grief group during an emotional Stroop task where individuals were tasked with ignoring deceased-related words (Arizmendi et al., 2016). The authors postulate that individuals experiencing complicated grief may be unable to emotionally regulate during reminders of the deceased. Additionally, nodes in the attention network are more active in participants with high avoidance of deceased-related thoughts during a mind-wandering task and were less likely to report thinking about the deceased (Schneck et al., 2019a,b). Avoiding reminders of the lost individual may be a maladaptive coping strategy for those who experience intense yearning and emotional pain resulting from such reminding. This may ultimately prolong the time it takes to heal from the loss as the individual has fewer opportunities to learn how to appropriately cope with these thoughts.

### Modeling Grief Using Social Loss in Pair-Bonded Animals

Several non-human mammal species form socially monogamous mating pairs, and such species have been used in a laboratory setting to study the formation and maintenance of strong social bonds. The prairie vole, in particular, has become a valuable tool for understanding the neural mechanisms underlying social relationships. Unsurprisingly, the OT and DA systems are involved in the pair bonding process, though additional work has described roles for vasopressin, the opioid system, and the serotonergic system (reviewed in McGraw et al., 2010). Recently, several vole research labs have begun to study the neural and behavioral effects of losing a pair bonded partner with the hopes of understanding more about how loss is processed in the brain. Bosch and colleagues found that male prairie voles that lost a female partner displayed increased stress responsivity compared to those that remained in an intact pair and those that were separated from a same-sex sibling (Bosch et al., 2009). This behavior also corresponded with increased corticosterone levels. Interestingly, they also found that pair-bonding regardless of loss resulted in increased CRH mRNA in the medial bed nucleus of the stria terminalis (mBST) and blocking central CRH receptors during loss reversed the increase in passive coping behavior of males separated from their partner. Other studies have repeatedly found that partner loss adversely affects stress-coping behavior and potentially does so through alterations in the physiological stress response (governed by the HPA axis). For example, separated males and females display higher corticosterone and ACTH circulation, higher resting heart rate, and an imbalance of autonomic nervous system function (McNeal et al., 2014). Sun and colleagues also found increased CRH, OT, and AVP immunoreactivity in the paraventricular nucleus (Sun et al., 2014). Moving downstream to assess receptor binding sites for the neuropeptides that appear to be affected by loss, Bosch et al. (2016) saw reduced OTR binding in the NAc shell of animals experiencing loss of a female partner compared to those experiencing loss of a male sibling. They then showed a direct influence of OT on stress-coping behavior by infusing either OT or an OTR antagonist into the NAc and saw a reduction and an increase, respectively, in passive coping behavior. Relationship quality also has a significant impact on distress following loss of a partner. Although the majority of prairie voles will form a partner preference with an opposite sex animal, a small percentage (∼25%) will not form a pair bond. These animals, termed non-bonded, do not show increased anxiety-like behavior following loss of their partner while pair bonded males are more anxious and more sensitive to pain after loss (Osako et al., 2018). This suggests that forming a strong, high-quality bond with another individual leads to more distress when that relationship is lost and corroborates findings in humans that suggest relationship quality and type influence grief outcome (Smigelsky et al., 2019; Sekowski and Prigerson, 2021).

A study in titi monkeys found that pair-bonding was associated with higher overall glucose uptake in the entire brain, and short-term separation from a pair-mate reduced glucose uptake in the ventral pallidum, lateral septum, PVN, and periaqueductal gray (PAG). Interestingly, long-term separation significantly reduced whole brain glucose uptake, suggesting reversal of the brain-wide changes in activity associated with a pair-bonded state (Hinde et al., 2016). Physiologically, short-term separation resulted in increased plasma cortisol concentrations, while long-term separated animals no longer showed heightened cortisol and instead had higher plasma OT concentrations. Elevated OT concentrations may be necessary for preserving social motivation for partner interaction during separation, as animals treated with an OT antagonist spent less time in proximity with their pair-mate upon reunion following a long separation (Cavanaugh et al., 2018).

Current research is attempting to determine a timeline for the degradation of bond-related neurochemistry. Male prairie voles that lose their partner will continue to display a preference for their partner for up to 2 weeks following loss (Sun et al., 2014). However, by 4 weeks post-loss this partner preference is no longer present, and these males are able to form new partnerships. In fact, 4 weeks of separation from an old partner is required to replace the preference for the first partner with the second partner (Harbert et al., 2020), supporting the theory that the brain adapts over time to the loss of a specific relationship. This research group followed these results with a study showing that pair bonding is associated with drastic changes in gene transcription in the NAc, many of which were degraded after 4 weeks of separation from a partner (Sadino et al., 2021). Future studies should assess whether variation in this process predicts individual differences in loss outcome as this could be a particularly valuable finding that is translational to humans coping with grief after losing a loved one.

While rats and mice do not display attachment to mates in the same way as socially monogamous animals, they do experience social attachment in the form of maternal behavior toward offspring. The maternal brain undergoes a host of neurochemical changes that enable high motivation, aggression toward intruders, and blunted emotional reactivity which all serve to create a behavioral phenotype that ensures survival of the infants (Lonstein et al., 2015). However, permanently removing pups from the mother reverses her low anxiety state (Kalyani et al., 2017), increases behavioral despair (Pawluski et al., 2009), and reduces social motivation and responsiveness of VTA DA neurons (Rincón-Cortés and Grace, 2021). Thus, postpartum social isolation, loss of the mother-infant social relationship, or some combination of both lead to similar behavioral and neurochemical phenotypes that are associated with isolation in other models. Additionally, maternal rats and mice are highly motivated to reunite with their offspring, as they will learn to bar press for access to their pups (Wilsoncroft, 1969; Lee et al., 1999) and even find them more rewarding than cocaine during early lactation (Mattson et al., 2003); thus, separation from infants results in motivation to reunite with them. Furthermore, infant loss in non-human primates can be devastating (Watson and Matsuzawa, 2018; Sharma et al., 2020), while infant loss in humans is associated with post-traumatic stress disorder (Jind et al., 2010), anxiety and depression (Kreicbergs et al., 2004; Hunter et al., 2017), and prolonged grief disorder (Lundorff et al., 2017). Therefore, utilizing rodent models of maternal separation to identify the possible neural underpinnings of loss and stress reactions during infant removal may prove to be beneficial for understanding parental grief reactions following infant death (for a recent review, see Demarchi et al., 2021).

## Summary and Conclusion

This review comes at a particularly salient moment where many individuals across the globe are experiencing isolation from peers and loved ones due to the COVID-19 pandemic. As such, loneliness has been prevalent over the last two years as people have been self-isolating or quarantining due to stay at home orders (Killgore et al., 2020; Li and Wang, 2020; van Tilburg et al., 2021), and even populations that are not typically at risk for feeling lonely (i.e., students) are suffering from consequences of isolation (Bu et al., 2020). While many individuals are experiencing a deficit in the quality and quantity of social relationships during the COVID-19 pandemic, a recent meta-analysis indicates that a variety of psychological interventions are successful in reducing feelings of loneliness (Hickin et al., 2021). However, another meta-analysis revealed that interventions that simply attempted to increase social support or social contact were less effective than those that attempted to target the maladaptive cognitions that foster loneliness (Masi et al., 2011), suggesting that individuals may need to take a more active role in breaking the cycle of loneliness. Thus far, no studies have examined brain activity or connectivity in lonely individuals that have received successful interventions. This would certainly be an interesting research topic, and we speculate that since interventions that are aimed at interrupting the negative thought cycle are more successful, this may be associated with increased cognitive control and thus an increase in activity within frontoparietal and/or cingulo-opercule regions.

Not only are we physically separated from much of our social support, but many people are also dealing with the loss of a loved one due to COVID-19. This shared global experience has emphasized the need for continued research on the neural underpinnings not only of loneliness, but of loss and grief processing as well. Like loneliness, loss of a loved one is associated with altered activity in similar brain regions, leading to alterations in motivation and stress reactivity. Although loneliness is a common experience for grieving individuals, research using humans and animal models suggest that the loneliness state during this period is unique from the experience of loneliness in the absence of loss. This is likely due to the structural and neurochemical changes that occur during the formation (and subsequent maintenance) of a strong bond/relationship that appears to be more rewarding, and therefore harder to get over, than isolation from peers. Further research using the prairie vole model of social loss appears to be aimed toward understanding the natural degradation of relationship-related neural pathways, while additional work should assess the features of an individual or a relationship that makes it harder to move on from some compared to others. Investigating loneliness, isolation, and loss through various lenses could allow us to find a new approach to improving the quality of life for those experiencing such conditions.

## Author Contributions

EV and AS wrote and edited all versions of this manuscript. Both authors contributed to the article and approved the submitted version.

## Conflict of Interest

The authors declare that the research was conducted in the absence of any commercial or financial relationships that could be construed as a potential conflict of interest.

## Publisher’s Note

All claims expressed in this article are solely those of the authors and do not necessarily represent those of their affiliated organizations, or those of the publisher, the editors and the reviewers. Any product that may be evaluated in this article, or claim that may be made by its manufacturer, is not guaranteed or endorsed by the publisher.
